# Validity and Reliability of the Indian Version of the HLS-EU-Q16 Questionnaire

**DOI:** 10.3390/ijerph18020495

**Published:** 2021-01-09

**Authors:** Jyoshma Preema Dsouza, Stephan Van den Broucke, Sanjay Pattanshetty

**Affiliations:** 1 Psychological Sciences Research Institute, Université catholique de Louvain, 1348 Louvain-la-Neuve, Belgium; stephan.vandenbroucke@uclouvain.be; 2 Prasanna School of Public Health, Manipal Academy of Higher Education, Manipal University, Manipal 576104, India; sanjay.pattanshetty@manipal.edu

**Keywords:** health literacy, India, measurement, validation

## Abstract

Health literacy is a key topic in public health. Several measurement tools exist that operationalize health literacy, but only a few standard tools measure health literacy at a population level, and none of those are currently available for the Indian context. This study aimed to develop and validate an Indian version of the short form of the European Health literacy Questionnaire (HLS-EU Q16). Following the translation of the English version of the questionnaire in Hindi and Kannada by language experts and confirmation of the item content by health literacy experts, the questionnaire was administered to 158 Hindi speaking and 182 Kannada speaking individuals, selected via purposive sampling. Pearson’s correlation was used to confirm test–retest reliability, and confirmatory factor analysis was used to assess the construct validity of the scales in both languages. Cronbach’s alpha was calculated for the scales and their sub-domains, and item-total correlations were used to calculate item discriminant indices. Discriminant validity was examined by comparing scores of participant groups based on educational status and training in health care. Cronbach’s alpha for the Hindi version of the tool (HLS-IND-HIN-Q16) was 0.98, and for Kannada version (HLS-IND-KAN-Q16) 0.97. Confirmatory factor analysis produced fit indices within acceptable limits. The results allowed us to conclude that the two Indian language questionnaires allow valid and reliable measurements of health literacy among the Hindi and Kannada speaking population of India.

## 1. Introduction

Health literacy (HL) has become a key concept in public health. While originally the term only referred to a person’s ability to understand medical information in a health care context, over the past two decades it has found its way into public health and has expanded in both scope and meaning [1]. Although different definitions are still being used, there is a growing consensus that HL refers to the cognitive and social skills that determine the motivation and ability of individuals to gain access to, understand, appraise and use information to promote and maintain good health [2,3]. As such, it includes functional skills that are required to access health information, such as reading and writing, as well as more advanced cognitive skills that allow us to understand this information, and in-depth cognitive and social skills that ultimately lead to better control of life events [4].

A large and consistently growing body of literature has demonstrated that low HL is associated with poor medication adherence, poor self-care management, less favorable treatment outcomes, lower participation in screening programs, suboptimal use of preventive services, lower engagement in health promoting behaviors, and a higher cost of care [1,5,6,7,8]. Furthermore, HL is increasingly recognized as a determinant and/or mediator of health inequalities [9,10]. It is therefore not surprising that a growing number of studies from across the world have looked at the prevalence of limited HL at the population level [11,12,13].

Contrary to the growing interest in HL in many countries, including several countries in Asia [13], and despite its key importance for public health and health care, HL has not yet been studied extensively in India. A few scholars have investigated the relationship of HL with neonatal, oral, or mental health in Indian samples [14,15,16,17], but (to date) no studies have been performed in India that measure HL at population level. This may partially be due to the lack of adequate measurement tools. The existing studies of HL in India typically involve the use of clinical interviews or screening tools such as the Rapid Estimate of Adult Literacy in Medicine (REALM), which only captures functional HL and are difficult to use on large samples or outside the clinical setting. There are numerous scales to measure health literacy. A systematic review reveals that most of the tools had a subjective or an objective measurement approach [18]. A tool applicable for a specific age group [19,20] or to measure clinical or functional literacy alone [21] cannot be used in population studies. Additionally, most of the tools are not available for use in other contexts. Well-validated tools to measure the broader concept of HL in the population exist and have been adapted and used in a wide range of contexts. The most widely used ones at international levels are the Health Literacy Questionnaire (HLQ), which measures nine dimensions of HL related to individual traits and abilities as well as contextual and health system resources [22], and the European Health Literacy Survey Questionnaire (HLS-EU-Q), which measures four dimensions of HL (i.e., access, understand, appraise and apply health information) across three domains (health care, disease prevention, and health promotion) [23,24]. This study validates the HLS-EU-Q tool because it measures health literacy across the four dimensions and across the domains of health care, disease prevention and health promotion. This tool has also been validated for use in several Asian countries [13]. A short version of the tool developed, comprises 16 of the 47 original items and has a high correlation with the original version. Besides, the HLS-EU-Q16 has been translated into more than 16 languages and is frequently used in population studies of HL, as it does not require much time to be completed, is easy to analyze, and can be used with people who have a low level of literacy [25].

None of the tools measuring the broader concept of HL at a population level have been translated or validated for any Indian language. The objective of this study was to translate the HLS-EU-Q16 into two languages of India, Hindi which is widely spoken and Kannada which is the regional language of Karnataka where the study was conducted, to evaluate its psychometric properties, including validity and reliability. About 43.6% of Indians (528,347,193 people) speak Hindi, and about 4.8% (43,706,512 people) speak Kannada [26].

## 2. Materials and Methods

### 2.1. Study Design and Setting

A cross-sectional, observational survey was performed among Hindi and Kannada speaking individuals in three geographically different states of India (Himachal Pradesh, Madhya Pradesh, and Karnataka) between 1 October 2019 and 30 November 2019.

### 2.2. Instrument

The HLS-EU-Q16 is a 16-item self-report questionnaire measuring difficulties experienced by the respondent in accessing, understanding, appraising, and applying information to tasks related to making decisions in health care, disease prevention, and health promotion. The items were selected from the original 47-item HLS-EU-Q47 based on Rasch Analysis [20] and represent 11 out of the 12 cells of the conceptual model that underlies the HL-EU-Q (i.e., four competences: access, understand, appraise and apply information, and three domains: health care, disease prevention, and health promotion). Items are rated on four-point Likert scales (1 = very difficult, 2 = difficult, 3 = easy, and 4 = very easy). To score the HLS-EU-Q16, the categories “very difficult” and “difficult” of each item are scored as 0, and the categories “easy” and “very easy” as 1, yielding a simple sum score ranging between 0 and 16. A score of 0–8 is considered as inadequate HL, a score between 9 and 12 as problematic, and 13 or more as sufficient. Alternatively, a standardized HL index score can be calculated by using the formula HL = (Average − 1) × (50/3), yielding a score on a scale of 50, with 0–25 considered as inadequate HL, 25–33 as problematic, 33–42 as sufficient, and higher than 42 as excellent. This study calculated the standardized HL index score to measure the level of health literacy of participants. The questionnaire had questions pertaining to participants’ age (years), gender (male or female), education (highest level of education acquired), training acquired as health care professional (yes or no), ability to afford medical expenses (4-point Likert scale of very difficult, difficult, easy or very easy) and self-perceived health status.

The original version of the HLS-EU-Q16 was translated from English into Hindi and Kannada by language experts, who also checked the translated items with Hindi and Kannada speaking individuals to assure readability. The translated versions were then back translated to English and compared to the original version by HL experts to verify similarity of the content. Each item was assessed for item content following the revision. Since none of the items had undergone a change of meaning in the translation, all were kept. The translated questionnaires were administered to 30 individuals who understood Hindi from Himachal Pradesh, Punjab, Rajasthan, Gujarat, Madhya Pradesh and 20 individuals who understood Kannada from Mangalore, Udupi, Bangalore, Mysore, Coorg, Tumkur, Chikmangalore, and Belgaum in Karnataka to check their ease of comprehension. Comments and observations made by the respondents were used to refine the wording of some items. To be able to use the tool for low-literacy individuals, participants who were unable to read but were able to understand the tool when read to them were included in the study.

### 2.3. Participants

The Hindi and Kannada versions of the questionnaire were administered to 158 Hindi speaking individuals and 182 Kannada speaking individuals, selected via a purposive sampling procedure among individuals who were present in public places or waiting lounges of a tertiary health care setting. Participants who were unable to read but who could understand the language were helped by the researchers, who read the questions out and noted the answers given by the participant.

Of the resulting participant samples, for the Hindi questionnaire, the ages varied between 25 and 65 years, with a median age of 35 years (IQR 25) and 85 (53.79%) of the participants were males. Fifteen (9.5%) individuals had not completed a high school level education, while only 4 participants (2.5%) had a graduate or a post graduate degree. Seven individuals (0.04%) were unable to read the tool and were assisted. In terms of socio-economic status, 62% of the participants asserted that they could easily or very easily afford health care expenditure. Forty-two (19.7%) had received a training in a health care related profession.

For the Kannada questionnaire, the median age of the participants was 32 (IQR 23). Half of them (*n* = 88, 48%) were males. Nineteen (10.4%) participants had not completed high school education, while 2 (1.1%) had a graduate or postgraduate degree. Eleven participants (0.06%) were unable to read the tool and were assisted. Of this sample, 59% participants claimed that they could easily or very easily afford health care expenditure, and only 25 (13.7%) had received training in a health care profession.

### 2.4. Analyses

Construct validity is the rational–empirical process that allows us to identify the psychological attributes of a measure or scale [27]. To establish the construct validity of the of Indian HLS-EU-Q16 scales, Confirmatory Factor Analysis (CFA) was performed, which verifies the causal relations among latent and observed variables in an a priori specified, theory-derived model. Specifically, CFA was applied to compare the structure obtained in both Indian subsamples to the three factors of “Health care”, “Disease Prevention” and “Health Promotion”, and the four subdomains “Access”, “Understand”, “Judge” and “Apply” information, as per the original model. To test the model fit, root mean square error of approximation (RMSEA), standardized root mean square residual (SRMR), comparative fit index (CFI), goodness of fit index (GFI) and adjusted goodness of fit index (TLI) were calculated. Item analysis was carried out by calculating item-total correlation coefficients in both subsamples, to measure the reliability of the questionnaire, test–retest reliability using Pearson correlations was checked in 10% of both the Hindi and Kannada subsamples, with re-testing taking place after one week, to ensure that the measurements are stable over time [28]. Internal consistency was checked via Cronbach’s alpha coefficient, which reflects the extent to which items within an instrument measure various aspect of the same characteristic or construct [29]. Cronbach’s alpha (α) greater than 0.8 proves good internal consistency [30]. Discriminant validity ensures that the measures of constructs that theoretically should not be highly related to each other, are not highly correlated to each other [31]. Standardized HL index scores were calculated for all participants, and mean scores of male and female participants, participants with high and low levels of education, and medically trained and non-trained participants were compared using an independent sample t-test to check discriminant validity. All analyses were conducted using the JASP Open-Source statistical software 2019 (JASP version 0.11.1.0, University of Amsterdam, The Netherlands).

### 2.5. Ethical Considerations

Prior to the study, ethical approval was obtained from the Father Muller Medical College Ethics Committee. All participants provided individual written consent.

## 3. Results

### 3.1. Test–Retest Reliability

Test–retest reliability after 1 week among the Hindi and Kannada subsamples gave Pearson’s correlation coefficients of *r* = 0.8 (*p* < 0.05) for the Hindi subsample, and *r* = 0.7 (*p* <0.05) for the Kannada subsample which show a high correlation.

### 3.2. Construct Validity

**Figure 1:**
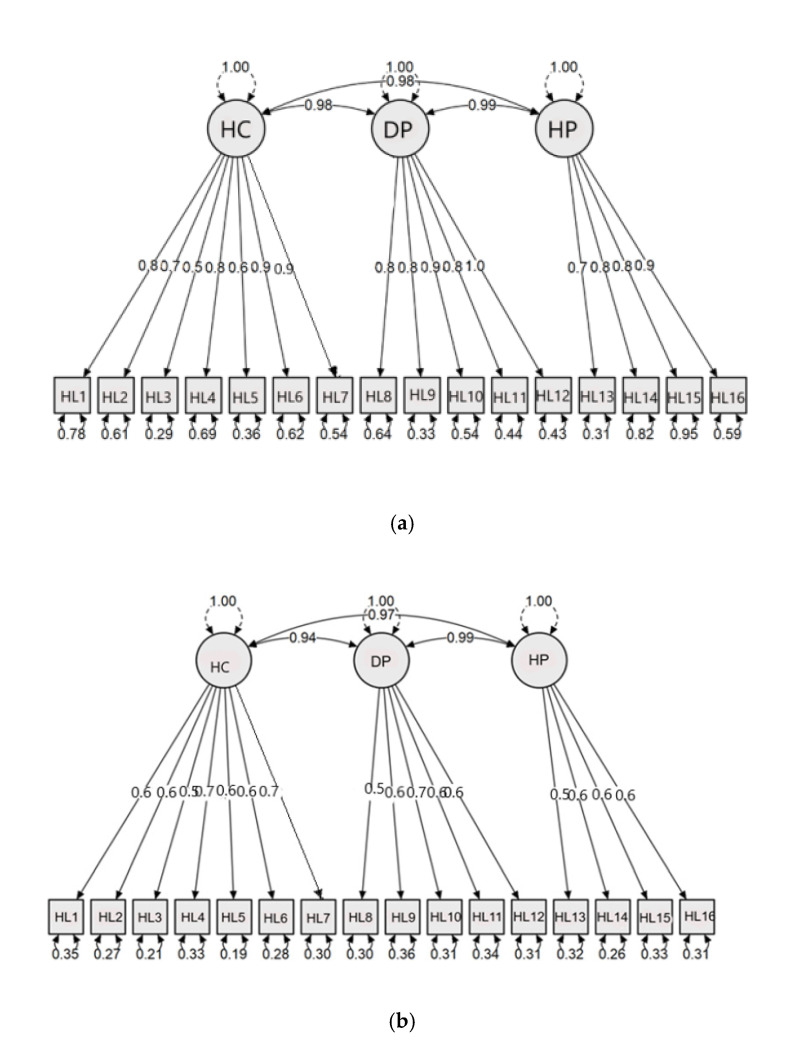
Measurement model and factor loadings for the Indian versions of the European Health Literacy Survey Questionnaire (HLS-EU-Q)16. (**a**) Hindi version (chi square (X^2^) = 123.884, df = 101, root mean square error of approximation (RMSEA) = 0.06). (**b**) Kannada version (chi square = 212.822, df = 101, RMSEA = 0.07). HC: Health Care; DP: Disease Prevention; HP: Health Promotion; HL1–16: Health Literacy measurement variable

To establish construct validity, confirmatory factor analysis was applied to consider the fit of the factor solution obtained in both Indian subsamples to the 3 factors of “Health care”, “Disease Prevention” and “Health Promotion” of the HLS-EU-Q16. The measurement model and factor loadings for each item for both subsamples are shown in Figure 1a,b. The fit indices for both subgroups are presented in Table 1. As this table indicates, the fit indices are within an acceptable range, with X^2^/d less than 5, and RMSEA < 0.08 indicating a good model fit. The (standardized) root mean square residual, which represents the square-root of the difference between the residuals of the sample covariance matrix and the hypothesized model, is <0.08. The CFI, which gives the fit of a target model to the independent, or null, model, is >90, and the TLI and GFI are both above 0.8. These indices allow us to conclude that the questionnaire has a very good construct validity [23].

### 3.3. Internal Consistency

The item-total correlation coefficients for the two Indian versions of the HLS_Q16 are given in Table 2. They vary from *r* = 0.44 (“understand information about unhealthy habits such as smoking, low physical activity or drinking too much alcohol” in the Kannada subsample) to *r* = 0.805 (“decide how you can protect yourself from illness using information from the mass media” in the Hindi subsample). All correlations are highly significant, thus confirming that all items contribute to the overall scale score. This is further confirmed by the Cronbach’s alpha coefficients, which are α = 0.95 and α = 0.92 for the Hindi and Kannada subsamples which are above the acceptable limits of >0.9, respectively. Removal of items did not increase the Cronbach’s alpha values in either sample.

### 3.4. Discriminant Validity

The standardized HL index scores for male and female participants, those with both high and low levels of education, medically trained and non-trained participants are shown in Table 3. As this table indicates, there was no significant difference in HL scores between males and females. In contrast, HL scores were significantly higher in individuals with a graduate or post graduate degree compared to those who had a lower degree or no education. Individuals trained in a health care profession also had higher HL scores than those who were not medically trained.

## 4. Discussion

Health literacy is recognized as a factor of importance in public health, in the sense that it enables people to find, understand, and process information that is required to make decisions that have an impact on their health [32]. To assess the level of HL in a population, it is necessary to use valid and comprehensive measures, that capture the different components of HL. Unlike many other countries, India thus far did not have a validated tool to measure HL at the population level in a comprehensive way. Existing studies involving health literacy in India typically used measurement tools that operationalize HL in a limited way. For instance, Cruz and Aradhya [17] and Haridas et al. [16] measured functional HL of patients, which is less comprehensive than recent definitions of the concept in that it does not include communicative and critical aspects. Furthermore, their tools are restricted to the health care sector and do not cover disease prevention and health promotion. Other tools used in India are concerned with specific forms of HL, like Kermode et al., [15] who measured mental HL in the community. The short version of HLS-EU-Q16 will thus be helpful to measure general HL in the Indian context.

The results of our study revealed that the Hindi and Kannada versions of the HLS-EU-Q16 are valid and reliable, with a very good construct validity replicating the dimensions and domains of the original English questionnaire, high levels of internal consistency, good test–retest reliability after 1 week, and relevant indications of discriminant validity achieved in both samples. These results are comparable or even better than those observed in other countries, with internal consistency coefficients for the HLS-EU-Q16 in Spain, France, Israel, Turkey, Italy, and Iceland all scoring above 0.799 [33,34,35,36,37,38] and the CFA indices across the three domains being similar to those reported in previously conducted studies [38]. All the fit indices obtained for our samples are within acceptable limits [39]

It is noted that the mean level of HL obtained by the participants in our study was low. This implies that a relatively large part of the population has limited or insufficient health literacy. However, large-scale population studies on health literacy such as the ones undertaken by National Assessment of Adult Literacy (NAAL) in the US, or the European Health Literacy Survey (HLS-EU), provide similar results. Moreover, no significant difference in HL score was observed in our samples between males and females. Other studies have arrived at similar results [34], although most population studies do reveal a significant difference in HL scores for gender [40,41].

On the other hand, there was a clear difference in HL scores, in the expected direction, between respondents who had been trained for a health-related profession and those who had not, which attests to the discriminant validity of the questionnaire.

Finally, our findings also revealed an effect of education, in the sense that participants who had completed secondary education or a higher level of education had higher HL scores than those who did not finish secondary education. This concurs with many other studies that found a positive association between education and HL [12,35,42]. It is noted that individuals who were unable to read but able to understand the language were included in our study by reading out the questions, to have a heterogenous sample. This shows the feasibility of using the questionnaire among low-literacy groups, which is a finding also reported by Storms et al. [25].

## 5. Limitations

The findings regarding the level of HL obtained in this study cannot be generalized. The study sample is not representative of the population as there was a high level of trained health care participants. To assess the level of health literacy in India, or in the states from which participants will be recruited, we suggest that the questionnaire be used on representative samples of the populations concerned. In addition, as the questionnaires were read to participants who were unable to read, there might have been a bias during data collection. So, while the two tools can be considered as valid and reliable and can thus be used by to measure HL both at individual and population levels, care should always be given to the recruitment of participants and to the way the questionnaires are used, depending on the purpose and context of the study.

## 6. Conclusions

The Hindi and Kannada versions of the HLS-EU-Q16 were found to be valid and reliable tools to measure HL for Hindi and Kannada speaking Indians. The tools can be used in future for educated and less educated individuals who speak the respective languages. As such, they can help researchers to measure HL both at an individual and at a population level.

## Figures and Tables

**Table 1:** Fit indices of measurement model for the Indian versions of the HLS-EU-Q16.

Compliance Criteria	Hindi Version		Kannada Version	
	Domain	Sub-Domains	Domain	Sub-Domain
X^2^/d	1.22	2.5	2.1	2.8
Standardized root mean square residual (SRMR)	0.061	0.05	0.074	0.07
Root mean square error of approximation (RMSEA)	0.06	0.07	0.07	0.08
Comparative Fit Index (CFI)	0.941	0.91	0.892	0.85
Tucker-Lewis Index (TLI)	0.927	0.9	0.881	0.81
Goodness of fit index (GFI)	0.806	0.9	0.873	0.82

d: Degrees of freedom of the model.

**Table 2:** Item total correlation co-efficient for the Kannada and Hindi versions of the HLS-EI-Q16.

HLS-Q16	Hindi Version	Kannada Version
To find information about symptoms of illnesses that concern you?	0.562	0.666
2. To find out where to get professional help when you are ill?	0.681	0.686
3. To understand what a doctor says to you	0.615	0.713
4. To understand your doctor’s or pharmacist’s instruction on how to take a prescribed medicine?	0.583	0.715
5. To judge if you may need to get a second opinion from another doctor?	0.749	0.712
6. To use information your doctor gives to you to make decisions about your illness?	0.687	0.703
7. To act on advice from your doctor or pharmacist?	0.652	0.791
8. To find information on how to handle mental health problems?	0.621	0.699
9. To understand information about unhealthy habits such as smoking, low physical activity or drinking too much alcohol?	0.435	0.775
10. To understand information about recommended health screenings or examinations?	0.628	0.744
11. To judge if the information on health risks in the mass media is reliable?	0.639	0.775
12. To decide how you can protect yourself from illness using information from the mass media?	0.686	0.805
13. To find information about activities that are good for your mental health and well-being?	0.638	0.781
14. To understand advice concerning your health from family or friends?	0.588	0.512
15. To understand information in the mass media on how to improve your health?	0.736	0.767
16. To judge which everyday habits affect your health?	0.584	0.743

**Table 3:** Standardized health literacy (HL) index scores for subgroups of participants.

Participant Groups	M	SD	*t*	*p* Value
**Gender**				
male	27.68	11.38	1.051	>0.05
female	25.66	11.40		
**Education Level**				
no secondary education (std 8th)	11.95	15.426	−5.258	<0.05 *
secondary education or above	28.69	9.704		
**Training in Health Care Profession**				
no training	25.28	10.05	−8.86	<0.01 **
training	43.43	6.144		

* Significant *p* < 0.05; ** significant *p* < 0.001.

## Data Availability

The data presented in this study are available on request from the corresponding author.
